# Influence of dietary protein on serum phosphorous levels in peritoneal dialysis patients with different initial transport function

**DOI:** 10.1080/0886022X.2022.2148537

**Published:** 2022-11-25

**Authors:** Xiao-Pei Wang, Ying Ma, Jing Lv, Yu Liang, Li Jin, Wan-Hong Lu, Chang-Na Liang, Bao Qian, Zhao Li

**Affiliations:** Department of Nephrology, The First Affiliated Hospital of Xi’an Jiaotong University, Xi’an, China

**Keywords:** Dietary protein intake, hyperphosphatemia, peritoneal dialysis, peritoneal transport function, peritoneal phosphate clearance

## Abstract

**Introduction:**

This cross-sectional study investigated the influence of dietary protein intake (DPI) on serum phosphate levels in peritoneal dialysis (PD) patients and determined the DPI cutoff required to prevent hyperphosphatemia.

**Methods:**

A total of 504 PD patients were categorized into fast (4 h dialysate/plasma [D/P] creatinine clearance ≥0.65) or slow (<0.65) peritoneal transporters. Serum phosphorus and peritoneal solute clearance were compared between the groups with different DPI.

**Results:**

The fast peritoneal transporters (*n* = 233) were older, had lower serum albumin and phosphorus levels, and had higher peritoneal phosphorus clearance (all *p* < 0.001). Among the slow transporters (*n* = 271), serum phosphorus levels were significantly higher among patients with DPI > 1.0 g/kg/d (*p* < 0.001). High DPI only increased the hyperphosphatemia risk in slow transporters (not in high transporters). DPI ≥1.026 g increased the hyperphosphatemia risk in those patients (area under the curve: 0.66, *p* = 0.001).

**Conclusion:**

High DPI increases the hyperphosphatemia risk in PD patients with slower peritoneal transport function.

## 1. Introduction

Hyperphosphatemia is a common complication in individuals undergoing dialysis, which is associated with a high incidence of cardiovascular events and poor outcomes [1]. Phosphate control interventions include dietary phosphorus restriction, the use of phosphorus binders, and adequate dialysis. The Kidney Disease Improving Global Outcomes and Kidney Disease Outcomes Quality Initiative guidelines recommend a daily protein intake of 1.2 g/kg of body weight [2]. High dietary protein intake (DPI) increases the risk for hyperphosphatemia and accelerates the loss of residual kidney function [3]. Dietary protein restriction is considered to be effective for controlling serum phosphorus levels [4]. However, low dietary protein worsens nutritional status and increases mortality in peritoneal dialysis (PD) patients [5]. The role of peritoneal phosphate clearance rate, an important indicator of phosphate balance, has been under appreciated in PD patients [6]. The peritoneal phosphate clearance rate is lower and serum phosphorus levels are higher in patients with low and average low peritoneal membrane function characteristics (slow peritoneal transporters) than in those with high and high average peritoneal membrane function characteristics (fast peritoneal transporters) [7]. Very few studies have investigated the effects of DPI on serum phosphate levels in PD patients with different peritoneal transport types.

This study investigated the effect of high protein intake on the incidence of hyperphosphatemia in PD patients with different peritoneal transport types.

## 2. Materials and methods

### 2.1. Study population

Newly catheterized PD patients undergoing regular follow-up at our PD center between January 2012 and December 2019 were enrolled in this study. Patients underwent a peritoneal equilibration test (PET) within 3 months of starting PD. None of the patients had peritonitis, tumors, other hypermetabolic states, or were undergoing corticosteroid treatment. All patients received a calcium content of 1.25% dialysate and were treated using a continuous ambulatory PD model. Patients treated using automated PD and day ambulatory PD were excluded.

### 2.2. Study methods

We reviewed the primary etiologies, sex, age, predialysis laboratory parameters, baseline estimated glomerular filtration rate (eGFR, calculated using the Chronic Kidney Disease Epidemiology Collaboration equation), PD adequacy at 1 month after starting dialysis, and clinical outcomes of the enrolled patients. The patients were classified as fast peritoneal transporters (4-h dialysate/plasma [D/P] creatinine clearance: ≥0.65) and slow transporters (4-h D/P creatinine clearance: <0.65). Standard parameters of dialysis adequacy were determined by measuring total Kt/V and liters of creatinine cleared by standard methods (L/week/1.73 m^2^)[8]. Residual GFR was calculated as the average of 24-h urinary urea and creatinine clearance.

The peritoneal solute clearance and urine solute clearances were calculated as follows:

$$\begin{array}{c} P\mathrm{eritoneal}\;\mathrm{phosphate}\;\mathrm{clearance}\;\left(L/\mathrm{week}/1.73\;m^{2}\right)=\left(\text{dialysate}\;\text{phosphate}\;\text{in}\;\text{mmol}/L/\text{plasma}\;\text{phosphate}\;\text{in}\;\text{mmol}/L\right)\\ \times 24-h\;\text{effluent}\;\text{dialysate}\;\text{volume}\;\left(L\right)\;\times \left(\text{corrected}\;\text{for}\;1.{73\;m}^{2}{\text{body}}^{}\text{surface}\;\text{area}\;\left[\text{BSA}\right]\right)\text{. [9]} \end{array}$$

$$\begin{array}{c} R\mathrm{enal}\;\mathrm{phosphate}\;\mathrm{clearance}\;\left(L/\mathrm{week}/1.73\;m^{2}\right)=\left(\text{urine}\;\text{phosphate}\;\text{in}\;\text{mmol}/d/\text{plasma}\;\text{phosphate}\;\text{in}\;\text{mmol}/L\right)\;\\ \times \;24-h\;\text{urinary}\;\text{volume}\;\left(L\right)\;\times \;\left(\text{corrected}\;\text{for}\;1.{73\;m}^{2}\text{BSA}\right)\text{. [9]} \end{array}$$

$$\mathrm{Peritoneal}\;\mathrm{protein}\;\mathrm{clearance}\;\left(\mathrm{Pcl}\right)\;\left(L/\mathrm{week}/1.73\;m^{2}\right)=24-h\;\text{dialysate}\;\text{protein}\;\text{loss}/\left(\text{serum}\;\text{albumin}/0.4783\right).$$

Pcl was expressed as mL of plasma cleared per day [10].

Compliance with the prescribed protein intake was assessed by monitoring 24-h urinary urea nitrogen levels according to the Mitch-Maroni equation [11]. The protein equivalent of nitrogen appearance (PNA) normalized to body weight was calculated using the methods described by Bergstrom et al. [12] The total 24-h peritoneal ultrafiltration (L/24-h) was recorded as the difference between the volume instilled and drained. In the steady-state, the normalized protein catabolic rate (nPCR) is equivalent to daily protein intake, which is normalized to weight (g/kg/day). This was estimated by the nPNA or nPCR, normalized to kilograms of body weight [13]. According to estimated DPI (eDPI) based on the nPCR at 1 month after starting PD, patients were divided into three groups: eDPI <0.80 g/kg/d (low-protein diet group, LPD Group), eDPI 0.8–1.0 g/kg/d (common protein diet group, CPD Group), and eDPI >1.0 g/kg/d (high proteins diet group, HPD Group). Patients with serum phosphate >1.78 mmol/L at 1 month after starting PD were defined as having hyperphosphatemia. Clearance was normalized to 1.73 m^2^ BSA. The impact of various DPI levels on serum phosphorus levels in patients with different peritoneal transport functions was compared. The amount of phosphorus binder at the first dialysis month and the baseline blood phosphorus values were collected.

### 2.3. Statistical analysis

Measurement data use the median and interquartile, count data use rate, as appropriate, and between-group differences were evaluated using the independent *t*-test and one-way analysis of variance. Non-normally distributed data were evaluated using the Kruskal–Wallis test. Multivariate analyses were undertaken with linear and binary logistic regression to establish a risk factor model of hyperphosphatemia and peritoneal phosphate clearance. Sex, age, peritoneal phosphorus clearance, urinary phosphorus clearance, GFR, total Kt/V, nPCR, ultrafiltration, urine volume, and dialysate dose were used as covariates in the binary logistic regression model. A receiver operating characteristic (ROC) curve was used to calculate the maximum cutoff value. Statistical significance was set at *p* < 0.05. All analyses were performed using IBM SPSS Statistics for Windows v.22.0 (IBM Corp., Armonk, NY, USA).

## 3. Results

### 3.1. Patient characteristics

A total of 504 PD patients were enrolled in this retrospective study. Baseline demographic and clinical characteristics are presented in Table 1. No significant differences in sex, baseline eGFR, predialysis serum phosphorus levels, urea levels, and creatinine levels were identified between fast and slow transporters. However, the fast peritoneal transporter group had a higher proportion of individuals with diabetes mellitus, older patients, and patients with lower serum albumin levels (*p* < 0.001).

**Table 1.** Baseline characteristics.

Characteristic	Total	Fast transporters	Slow transporters	p-value
Sex: male (n, %)	288/504 (57.1%)	155/288 (53.8%)	133/288 (46.2%)	0.176
Age, years (mean ± SD)	47.77 ± 15.16	50.38 ± 14.75	45.53 ± 15.18	<0.001
Primary disease				
Diabetes mellitus	95 (18.8%)	60/95 (63.2%)	35/95 (36.8%)	<0.001
Glomerular nephritis	338 (67.1%)	146/388 (37.6%)	192/399 (62.4%)	
Hypertension/interstitial nephritis	57 (11.3%)	25/57 (43.9%)	32/57 (56.1%)	
Vasculitis	14 (2.8%)	2/14 (14.3%)	12/14 (85.7%)	
eGFR at the start of PD mL/min/1.73m^2^ (mean ± SD)	6.71 ± 3.58	6.81 ± 3.95	6.62 ± 3.22	0.569
Blood biochemistry before dialysis				
Serum urea, mmol/L (mean ± SD)	29.25 ± 11.48	30.07 ± 12.49	28.57 ± 10.50	0.148
Serum creatinine, μmol/L (mean ± SD)	820.50 ± 304.95	832.41 ± 342.51	811.58 ± 265.41	0.453
Serum albumin, g/L (mean ± SD)	34.48 ± 5.69	33.06 ± 5.66	35.71 ± 5.43	<0.001
Serum phosphorus,mmol/L（mean ± SD）	1.83 ± 0.63	1.82 ± 0.69	1.83 ± 0.56	0.813

eGFR, estimated glomerular filtration rate; PD, peritoneal dialysis; SD, standard deviation.

Data obtained during the initial month after starting PD are shown in Table 2. The mean 4-h D/P creatinine clearance was higher in fast peritoneal transporters than in slow transporters (*p* < 0.001). Fast peritoneal transporters had higher systolic blood pressure (*p* < 0.001) and lower ultrafiltration volumes (*p* < 0.001). There were no differences in nPCR, urine volume, dialysis adequacy indices, and residual renal function between fast and slow transporters.

**Table 2.** Comparison of baseline values in the initial dialysis month between fast and slow transporters.

	Fast transporters	Slow transporters	p-value
	(*n* = 233)	(*n* = 271)	
Age (years)	50.38 ± 14.75	45.53 ± 15.18	<0.001
4-h D/P cr	0.75 ± 0.07	0.54 ± 0.07	<0.001
SBP (mmHg)	140 (120, 150)	130 (120, 140)	<0.001
DBP (mmHg)	80 (80, 90)	80 (71, 90)	0.010
Dialysis dose (mL)	6978.54 ± 1233.32	6798.71 ± 1137.12	0.091
Urine volume (mL)	890.39 ± 468.11	830.86 ± 425.40	0.137
Ultrafiltration (mL)	450.88 ± 203.19	628.45 ± 449.52	<0.001
nPCR (g/kg/d)	0.94 ± 0.19	0.96 ± 0.21	0.202
Total Kt/V	2.16 ± 0.55	2.15 ± 0.59	0.842
GFR (mL/min/1.73m^2^)	3.73 ± 2.66	3.79 ± 2.84	0.799

Data are presented as mean ± standard deviation, medians and quartiles.

D/Pcr, dialysate/plasma creatinine; SBP, systolic blood pressure; DBP, diastolic blood pressure; nPCR, normalized protein  catabolic rate; Kt/V, weekly urea clearance; GFR, glomerular filtration rate.

Phosphorus binders were used in 160 slow transporters and 50 fast transporters. The daily dosages of calcium-based binders in 46 slow transporters were higher than those in 17 fast peritoneal transporters (*p* = 0.009). The daily dosages of noncalcium based binders in 114 slow transporters were higher than those in 33 fast peritoneal transporters (*p* < 0.001). The dosage of the different phosphorus binders were converted and presented as the relative phosphate-binding coefficient (RPBC) in Table 6 [26].

### 3.2. Biochemical indexes and phosphorus clearance

**Figure 1.**
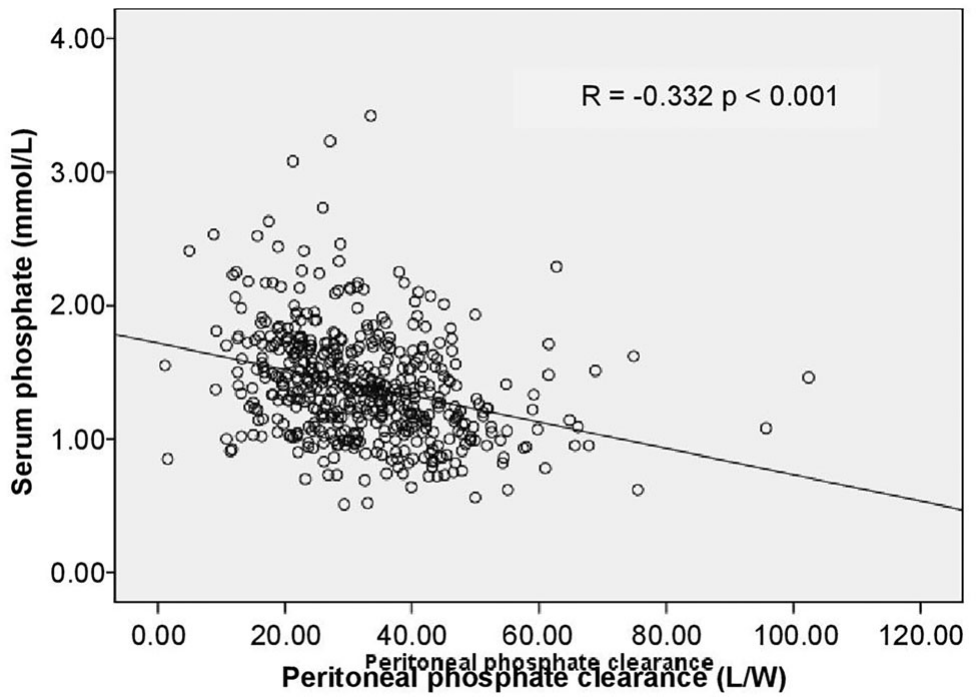
Influence of peritoneal phosphate clearance on serum phosphate levels in peritoneal dialysis patients.

Compared with slow transporters, fast peritoneal transporters had lower serum urea (*p* = 0.003), creatinine (*p* = 0.016), albumin, uric acid (*p* < 0.001), and hemoglobin (*p* = 0.003) and greater peritoneal protein clearance (*p* < 0.001) (Table 3). Although there were no differences in baseline serum phosphorus levels, phosphorus binder dosage, and dialysis dose, the serum phosphorus levels in the initial dialysis month were significantly lower in fast peritoneal transporters than in slow transporters (*p* < 0.001). Peritoneal phosphorus clearance and serum phosphorus levels were negatively correlated (*r* = 0.332, *p* < 0.001; Figure 1). Fast peritoneal transporters had greater peritoneal phosphorus clearance (*p* < 0.001) but lower urine phosphorus clearance (*p* = 0.037) than slow transporters. Daily PD phosphorus removal was greater in fast peritoneal transporters (*p* < 0.001). Nevertheless, total daily urine phosphorus removal was higher in slow transporters (*p* = 0.001). Total daily phosphorus removal rates of the two transporter groups were similar (*p* = 0.818; Table 3).

**Table 3.** Comparison of biochemical indicators and phosphorus removal status of different peritoneal transporter types in the initial dialysis month.

	Fast transporters (*n* = 233)	Slow transporters (*n* = 271)	p-value
Hemoglobin (130-175 g/L)	103.49 ± 17.18	108.20 ± 18.39	0.003
Serum urea (3.6-9.5 mmol/L)	17.16 ± 5.11	18.56 ± 5.55	0.003
Serum creatinine (57-111 μmol/L)	648.19 ± 234.75	699.0 ± 123.60	0.016
Serum uric acid (208-428 μmol/L)	350.48 ± 74.00	409.85 ± 94.69	<0.001
Glucose (3.9-6.1 mmol/L)	5.45 ± 1.80	5.40 ± 2.03	0.759
Serum albumin (40-55 g/L)	34.28 ± 5.11	38.50 ± 4.66	<0.001
Parathyroid hormone (15-65 pg/mL)	325.79 ± 248.30	372.44 ± 286.33	0.058
Peritoneal protein clearance (L/week)	83.92 ± 45.54	53.27 ± 24.40	<0.001
Serum phosphorus (0.81-1.45 mmol/L)	1.29 ± 0.36	1.48 ± 0.42	<0.001
Serum phosphorus at the start of PD (mmol/L)	1.81 ± 0.69	1.83 ± 0.55	0.816
Total daily urinary phosphorus removal (mmoL)	3.82 ± 3.06	4.83 ± 3.51	0.001
Daily PD phosphorus removal (mmoL)	6.30 ± 2.49	5.37 ± 2.34	<0.001
Total daily phosphorus removal (mmoL)	10.12 ± 4.21	10.21 ± 4.26	0.818
Urinary phosphorus clearance (L/week)	21.74 ± 16.83	24.93 ± 16.87	0.037
Peritoneal phosphorus clearance (L/week)	36.59 ± 12.17	29.15 ± 14.31	<0.001

Data are presented as mean ± standard deviation.

PD: peritoneal dialysis.

### 3.3. Effect of protein intake on serum phosphorus levels in PD patients

In fast transporters, high protein intake had no significant effect on serum phosphorus levels; however, the hyperphosphatemia risk was significantly elevated in slow transporters with an eDPI of more than 1.0 g/kg (Table 4). Nonetheless, among all patients and among slow transporters, serum phosphate levels were higher in HPD Group than in CPD Group (*p* = 0.033) and LPD Group (*p* = 0.006). In fast transporters, no significant differences in serum phosphorus levels were identified among different eDPI groups (Table 4).

**Table 4.** Influence of eDPI on serum phosphorus levels in different peritoneal transporter types.

Serum phosphorus	LPD Group	CPD Group	HPD Group	P value
(mmol/L)	(<0.80 g/kg/d)	(0.81‒1.0 g/kg/d)	(>1.0 g/kg/d)	
All patients	1.26 ± 0.39^**Δ^	1.38 ± 0.38*	1.47 ± 0.43^Δ^	<0.001
Slow transporters	1.32 ± 0.43**	1.42 ± 0.38**	1.62 ± 0.42^ΔΔ^	<0.001
Fast transporters	1.22 ± 0.34	1.33 ± 0.38	1.28 ± 0.36	0.181

Data are presented as mean ± standard deviation.

Compared with HPD Group: *p < 0.05, **p < 0.01; compared with CPD Group: ^Δ^*p* < 0.05, ^ΔΔ^*p* < 0.01.

eDPI: estimated dietary protein intake.

### 3.4. Factors influencing peritoneal phosphate clearance in PD patients

Linear logistic regression showed that peritoneal phosphate clearance was negatively associated with serum phosphorus levels (*p* < 0.001), GFR (*p* < 0.001), and urinary phosphorus clearance (*p* = 0.001) but was positively associated with 4-h creatinine D/P assessed by PET (*p* < 0.001), peritoneal protein clearance (*p* = 0.009), total peritoneal creatinine clearance (*p* < 0.001), total peritoneal urea clearance (*p* < 0.001), and dialysis dose (*p* < 0.001) (Table 5).

**Table 5.** Results of logistic linear regression for the identification of factors influencing peritoneal phosphorus clearance.

	β	t	P	95% CI
Serum phosphorus	−0.478	−5.093	<0.001	−0.662 to −0.293
GFR	−1.494	−5.738	<0.001	−2.005 to −0.982
Urinary phosphorus clearance	−0.010	−3.460	0.001	−0.005 to −0.016
Peritoneal protein clearance	0.002	2.613	0.009	0.001–0.004
Total peritoneal urea clearance	0.375	4.770	<0.001	0.220–0.529
Total peritoneal creatinine clearance (L/W)	0.020	8.175	<0.001	0.016–0.025
Dialysis dose (mL)	0.845	12.240	<0.001	0.709–0.980
PET 4-h D/Pcr	1.872	6.695	<0.001	1.323–2.422

CI: confidence interval; eDPI, estimated dietary protein intake; GFR, glomerular filtration rate; D/Pcr, dialysate/plasma creatinine.

### 3.5. Factors influencing serum phosphorus levels in different peritoneal transporter types

The incidence of hyperphosphatemia was 13.4% (*n* = 68) in all the patients, including 69.1% in slow transporters, which was significantly higher than 30.9% in fast peritoneal transporters (*p* = 0.001). The incidence of hypophosphatemia, which was defined as a serum phosphorus concentration of less than 0.81 mmol/L, was 4.1% (21/504). It was more common in fast peritoneal transporters (76.2% vs. 23.8%, *p* = 0.001). According to the univariate analysis, younger age, male sex, lower dialysis dose, poor GFR, insufficient urinary phosphorus clearance, peritoneal phosphorus clearance, higher nPCR, and lower total Kt/V were associated with hyperphosphatemia. According to the multivariate logistic regression analysis, only peritoneal phosphate clearance remained statistically significant in all patients (Table 6). Lower GFR (odds ratio [OR] = 0.475 and *p* = 0.034) and urinary phosphorus clearance (OR = 0.096 and *p* = 0.005) and nPCR more than 1.0 g/kg (compared with 0.8–1.0 g/kg: OR = 0.291 and *p* = 0.031; compared with <0.8 g/kg: OR = 0.100 and *p* = 0.013) were independent risk factors for hyperphosphatemia in slow transporters. The area under the ROC curve of nPCR in slow transporters with hyperphosphatemia was 0.66 (95% confidence interval: 0.577–0.742) and indicated that nPCR = 1.026 g/kg/day was the threshold.

**Table 6.** Results of binary logistic regression for the identification of risk factors for hyperphosphatemia in different peritoneal transporter types.

	Fast peritoneal transporters (*n* = 233)				Slow transporters (*n* = 271)			
	Β	P value	Exp (B)	95% CI	β	P value	Exp (B)	95% CI
Male	1.503	0.142	0.222	0.030-1.653	0.198	0.752	1.218	0.357-4.156
Age	-0.020	0.393	0.981	0.938-1.026	-0.033	0.062	0.968	0.935-1.002
Peritoneal phosphorus clearance	-0.072	0.010	0.930	0.881-0.983	-0.088	0.015	0.916	0.854-.983
GFR (mL/min/1.73m^2^)	-0.667	0.063	0.513	0.254-1.037	-0.745	0.034	0.475	0.239-.944
Urinary phosphorus clearance	0.041	0.370	1.041	.953-1.138	-0.051	0.005	0.096	0.018-.499
nPC*R* > 1.0 g/kg/d					7.286		1	
0.8–1.0 g/kg/d	-0.614	0.454	0.541	0.109-2.698	-1.235	0.031	0.291	0.095-0.891
<0.8 g/kg/d	-0.717	0.448	0.488	0.077-3.106	-2.302	0.013	0.100	0.016-0.618
TKt/V	-1.216	0.365	0.297	0.021-4.125	-0.088	0.107	0.622	0.349-1.108
Dialysis dose (mL)	0.001	0.827	1.000	0.999-1.001	0.001	0.084	1.001	1.000-1.001
Relative phosphate binder（RPBC）	0.018	0.955	1.018	0.550-1.884	-0.377	0.126	0.686	0.423-1.112

CI: confidence interval; GFR, glomerular filtration rate; nPCR, normalized protein catabolic rate; TKt/V, total weekly urea clearance.

## 4. Discussion

Hyperphosphatemia is an independent risk factor for vascular calcification, cardiovascular events, and all-cause mortality in PD patients [10]. Dietary phosphorus restriction is considered an effective and essential measure for lowering elevated serum phosphorus levels [8,13]. However, despite dietary phosphate restriction and phosphate binder use, serum phosphate levels remain significantly high in approximately 65% of the dialysis population [14,15]. On the other hand, severe dietary phosphorus restriction, without dietitian guidance, may result in insufficient intake of other nutrients, including protein and calories, [16] which is associated with malnutrition in dialysis patients [4,14]. This study found that fast transporters may counteract the effects of a high-protein diet on serum phosphorus level due to the higher phosphorus clearance. However, a significant increase in serum phosphorus levels was observed in HDP group those who with slower transport status. Therefore, recommending a different DPI for PD patients with different transport function statuses is necessary and may be more favorable for serum phosphorus control.

Peritoneal phosphorus clearance helps maintain phosphorus balance [5]. The molecular weight of phosphate (96 Da) is intermediate to that of urea (60 Da) and creatinine (130 Da), whereas the molecular radius of phosphate (2.8 Å) is closer to that of creatinine (3.0 Å) than that of urea (1.8 Å) [17]. However, phosphate has hydrophilic properties and may behave like a larger molecule in terms of solute clearance which diffuses more slowly. Because peritoneal phosphate clearance is time-dependent, it may be altered by differences in peritoneal membrane transport characteristics [18]. Our data indicate that higher 4-h D/P creatinine clearance in PET is associated with a higher peritoneal phosphorus clearance rate and lower serum phosphorus levels (Table 5), as shown previously [5,8]. Hyperphosphatemia seems to be more prevalent among slow transporters because of insufficient peritoneal phosphorus removal [19]. In this study, 69.1% of slow transporters suffered from hyperphosphatemia, which was much higher than the rate in the fast transporters.

Our data indicated no difference in baseline serum phosphorus levels, phosphorus binder dosage, and dialysis dose; however, serum phosphorus levels in the initial dialysis month were significantly lower in fast peritoneal transporters than in slow transporters. That may be due to the higher peritoneal phosphorus clearance rate (Figure 1). Despite peritoneal phosphorus clearance rate in slow peritoneal transports is weak, urinary phosphorus clearance was significantly higher than that in fast peritoneal transporters though the GFRs of the two groups in the initial dialysis month were similar (Tables 2, 3). These results indicated that, early on, urinary and peritoneal phosphorus clearances were complementary during PD. For patients with better residual renal function, urinary phosphorus clearance plays an important role in regulating the phosphorus balance, particularly in patients with slower peritoneal transport function (Table 6); Thus, retaining residual renal function is essential for serum phosphorus control [20,21].

According to our data, the albumin levels were significantly lower in fast peritoneal transporters than in slow transporters due to greater peritoneal protein clearance (*p* < 0.001, Table 3), and increasing the needs for protein supplementation to maintain serum albumin levels.Though a higher risk of hypoproteinemia and poor nutritional status, [21] there was no difference in serum phosphorus levels among the three eDPI groups in fast peritoneal transporters (Table 4). This means that peritoneal phosphorus clearance was sufficient to address the phosphorus load due to high dietary protein in the fast transporters [22]. Therefor, a high DPI to improve hypoalbuminemia did not increase the hyperphosphatemia risk in fast peritoneal transporters (Table 6).

According to the 2020 Clinical Practice Guidelines for Nutrition in Chronic Kidney Disease, protein intake of 1.0–1.2 g/kg/day in PD patients to supplement the protein loss in the dialysate is required [23]. According to our data, an eDPI >1.0 g/kg is a risk factor for hyperphosphatemia in slow transporters (Table 6) and may be associated with insufficient peritoneal phosphorus clearance. Serum phosphate levels in the HPD Group were higher than those in the other groups (*p* < 0.001; Table 3). Moreover, slow transporters had better albumin levels than fast transporters, and it follows that there is no necessary for slow transporters to overmuch daily protein intake to maintain serum albumin levels. In contrast, blood phosphate retention is largely due to high DPI in these patients. Our study showed that both LPD Group and CPD Group significantly reduce risks of hyperphosphatemia compared with HPD Group in slow transporters. On the other hand, a higher urinary phosphorus clearance rate was linked to lower serum phosphorus levels in slow transporters (Table 6). For these patients, high protein intake leads to accelerated loss of residual renal function [3] combined with decreased urinary phosphorus clearance, which weakens the compensatory effect of the kidney on phosphorus removal. According to our study, for patients with slower peritoneal transport function, the optimal daily protein intake recommendation of 1.026 g/kg/d could minimize hyperphosphatemia risk. Unfortunately, peritoneal transport function is typically not considered during dietary education in the real world.

For slow transporters, personalized diets with reduced protein intake should be recommended unless hypoproteinemia has already occurred. However, in our date, there were trends to nutritional status worsen in the LPD than HPD groups who with slower transport function. Therefore, dietary protein intake should not lower than 0.8 g/kg/d in these patients. The key to serum phosphorus control is intervention by a nutritionist who could recommend proteins with a low phosphorus:protein ratio, [24] such as egg white, and foods with the appropriate carbohydrates and fats, and this may have a bearing on survival. According to the patient’s dietary habits, a phosphorus binder should be used during one or two meals per day to prevent an increase in serum phosphorus levels [25]. Personalized low-phosphorus diet recommendations may aid to avoiding the rapid loss of residual renal function due to high protein intake and in retaining the compensatory effects of urinary phosphate clearance [14]. High-phosphorus foods are related to cooking methods and smoky, bake food (maken by egg yolk or cream), convenience food, snack and processed food, which are widely existed in Chinese and Western diet. Therefore, dietitians are very necessary to direct the food cooking for patients.

In addition, ultrafiltration was significantly less in fast peritoneal transporters compared with slow transporters in our study (Table 2). The amount of peritoneal ultrafiltration does not affect the amount of peritoneal phosphate removal. Phosphorus transport depends on dispersion instead of convection [18]. Longer dwell times may help control hyperphosphatemia in slow transporters [20]. In addition, total phosphate removal could be increased by increasing the total dialysis dose in fast peritoneal transporters with hyperphosphatemia (Table 5). Phosphate control can be facilitated by regulating the peritoneal prescription according to differences in peritoneal membrane transport status, residual renal function, and DPI in PD patients [19].

This was a single-center cross-sectional study and only studied the influence of dietary protein on serum phosphorous levels in peritoneal dialysis patients with different initial transport function at 1 month after PD is started. Thus, the conclusion of this study may not be generalizable to the long-term effects. It is necessarily to reassess the transport status after long dialysis duration to see whether the phosphate level in slow and fast transporter status would be influenced in different protein dietary groups. Since the DPI was evaluated by nPCR, actual values of dietary protein and phosphorus intake are needed to confirm the conclusions of this study. Finally, some other variables such as vitamin D analogue use, PD modality, dialysis and phosphorus binder adherence which may play into serum phosphorus are needed to considered in research design and statistical analysis. In the future, prospective multi-center clinical studies should be designed to verify the effect of a high protein diet on blood phosphorus in patients with long-term dialysis, peritoneal sclerosis, or recurrent peritonitis and to confirm the conclusions of this study.

In summary, high dietary protein (nPCR >1.026 g/kg/day) increased the hyperphosphatemia risk in PD patients with slow peritoneal transport function. However, it had little effect on patients with a faster status. Individualized protein dietary recommendations and treatment strategies for hyperphosphatemia in PD patients should be considered according to different peritoneal transport types, and this may lead to better control of serum phosphorus levels.

## Data Availability

All data generated or analyzed during this study are included in this published article.
